# Quenched lattice fluctuations in optically driven SrTiO_3_

**DOI:** 10.1038/s41563-023-01791-y

**Published:** 2024-02-01

**Authors:** M. Fechner, M. Först, G. Orenstein, V. Krapivin, A. S. Disa, M. Buzzi, A. von Hoegen, G. de la Pena, Q. L. Nguyen, R. Mankowsky, M. Sander, H. Lemke, Y. Deng, M. Trigo, A. Cavalleri

**Affiliations:** 1 Max Planck Institute for the Structure and Dynamics of Matter, Hamburg, Germany; 2 Stanford Pulse Institute, SLAC National Accelerator Laboratory, Menlo Park, CA USA; 3 School of Applied & Engineering Physics, Cornell University, Ithaca, NY USA; 4 Linac Coherent Light Source, SLAC National Accelerator Laboratory, Menlo Park, CA USA; 5 Paul Scherrer Institut, Villigen, Switzerland; 6 Department of Physics, Clarendon Laboratory, University of Oxford, Oxford, UK

**Keywords:** Phase transitions and critical phenomena, Nonlinear phenomena, Terahertz optics, Ultrafast photonics, X-rays

## Abstract

Crystal lattice fluctuations, which are known to influence phase transitions of quantum materials in equilibrium, are also expected to determine the dynamics of light-induced phase changes. However, they have only rarely been explored in these dynamical settings. Here we study the time evolution of lattice fluctuations in the quantum paraelectric SrTiO_3_, in which mid-infrared drives have been shown to induce a metastable ferroelectric state. Crucial in these physics is the competition between polar instabilities and antiferrodistortive rotations, which in equilibrium frustrate the formation of long-range ferroelectricity. We make use of high-intensity mid-infrared optical pulses to resonantly drive the Ti–O-stretching mode at 17 THz, and we measure the resulting change in lattice fluctuations using time-resolved X-ray diffuse scattering at a free-electron laser. After a prompt increase, we observe a long-lived quench in R-point antiferrodistortive lattice fluctuations. Their enhancement and reduction are theoretically explained by considering the fourth-order nonlinear phononic interactions to the driven optical phonon and third-order coupling to lattice strain, respectively. These observations provide a number of testable hypotheses for the physics of light-induced ferroelectricity.

## Abstract (web summary)

Intense light pulses can induce symmetry breaking, as for the generation of ferroelectricity in SrTiO_3_. Using ultrafast X-ray diffuse scattering at a free-electron laser, nonlinear phonon interactions that occur on such mid-IR excitation are observed, with a theory for the dynamics presented.

## Main

Functionally relevant ferroic order in quantum materials can be manipulated by coherently driving the lattice with optical and terahertz (THz) pulses^1–12^. New physical phenomena and non-equilibrium phases that have no equilibrium counterpart have been discovered following these protocols. The underlying structural dynamics have been mostly studied by recording the average atomic position along dynamical structural coordinates with elastic scattering methods^13–20^. The role of crystal lattice fluctuations in these optically driven dynamics, however, have remained largely unexplored^21^.

**Fig. 1:**
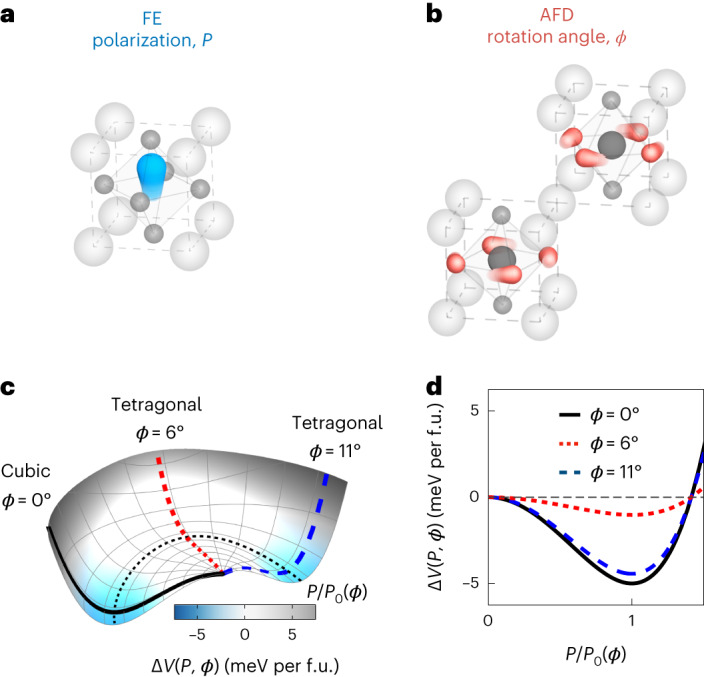
Fundamental distortions of STO. **a**, Polar distortion, creating the symmetry-broken FE state with polarization *P*, involves the displacement of the centre Ti atom along the *c* axis. **b**, AFD distortion involves the rotation of the oxygen octahedra around the *c* axis by angle *ϕ*, with a (1/2 1/2 1/2) wavevector. **c**, Illustration of the change in potential energy Δ*V*(*P*, *ϕ*) = *V*_TOT_(*P*, *ϕ*) – *V*_TOT_(0, *ϕ*) along the FE coordinate *P* for a range of AFD rotation angles *ϕ*, with *V*_TOT_(*P*, *ϕ*) being the DFT total energy, which is not the same for all the *ϕ*-dependent *P* = 0 states. As Δ*V*(*P*, *ϕ*) measures the difference between two states, it is zero for all the angles at the origin with *P* = 0, although the structures are different. Starting from *ϕ* = 0°, the depth of the potential energy Δ*V* along *P* reduces with the onset of AFD rotations. At *ϕ* = 6°, which is the rotation angle of the equilibrium tetragonal state, the gain in potential energy Δ*V*(*P*_0_, 6°) is reduced by a factor of five compared with Δ*V*(*P*_0_, 0°). Rotations beyond this angle revive the deeper instability of the FE state. **d**, Cuts of potential energy Δ*V*(*P*, *ϕ*) along the FE coordinate *P* for selected rotation angles *ϕ*. Source data

Here we focus on the cubic perovskite structure, which is inherently unstable, frequently undergoing different types of symmetry-breaking structural distortion. SrTiO_3_ (STO) is an especially interesting case, in which both ferroelectric (FE) and antiferrodistortive (AFD) instabilities (Fig. 1a,b) compete to determine the ground state. The cubic structure appears to be primed for an FE transition, which is expected on cooling since the polar distortion is the dominant symmetry breaking change. However, ferroelectricity fails to materialize, because below 110 K, an AFD instability induces a low-symmetry tetragonal phase. Although large polar fluctuations set in below 30 K, resembling a precursor for the FE phase transition, long-range polar order is stifled by zero-point fluctuations. This phenomenon is sometimes referred to as quantum paraelectricity^22,23^. The frustrated state of matter can be lifted by either the isotope replacement of oxygen^24^, partial cation doping^25^ or epitaxial strain^26^, which trigger the formation of a macroscopic FE polarization.

These physics are discussed in quantitative detail^27^ in Fig. 1c,d, where we report the results of ab initio calculations. Starting from the cubic structure (AFD rotation angle *ϕ* = 0°), the potential energy along the FE coordinate *P* develops a distinct minimum at a finite polarization *P*_0_, indicating the existence of an FE instability. When the AFD rotations at the (1/2 1/2 1/2) wavevector set in, the potential minimum at the *ϕ*-dependent polarization *P*_0_ along *P* becomes shallower. In the vicinity of *ϕ* = 6°, which is the calculated equilibrium value of the tetragonal phase, the condensation into the FE phase becomes substantially suppressed, as the depth of potential energy at *P*_0_ is comparable with the amplitude of zero-point fluctuations of the polar soft mode. A further increase in this AFD rotation to *ϕ* = 11° would revive the deeper FE instability, with a potential minimum similar in size to that of the cubic state at *ϕ* = 0°. Hence, the quantum paraelectric state of STO with suppressed ferroelectricity exists only in the low-temperature tetragonal structure for a narrow range of AFD rotation angles near *ϕ* = 6°. Note that experimentally the absolute rotation angle may be slightly different from this value, although the physical picture remains the same.

Intense THz and mid-infrared (mid-IR) light pulses were shown to remove this frustration and to induce ferroelectricity^9,10^, either transiently when directly coupling to the soft mode^10^ or permanently when a higher-frequency auxiliary mode was driven^9^. In the latter case, it was argued that the coupling of the resonantly driven Ti–O stretch phonon to acoustic modes may produce a strain field that stabilizes a long-range polar phase. Although the dynamics of the AFD mode are also expected to contribute, their role in these physics has not been established.

## Time-resolved X-ray diffuse scattering

Here we used time-resolved X-ray diffuse scattering^28^ to experimentally map the AFD lattice fluctuations at the Brillouin-zone boundary (1/2 1/2 1/2) R point in the cubic high-temperature phase of STO. The experiments were performed on a (100)-oriented STO substrate, cooled to a lattice temperature of 135 K, at the Bernina endstation of the SwissFEL^29^ free-electron laser. The sample was excited by mid-IR pulses of ~150 fs duration with a central frequency of 18 THz, bandwidth of 5 THz (full-width at half-maximum) and fluence of up to 60 mJ cm^–2^. The excitation spectrum covered the 17 THz transverse-optical resonance and the Reststrahlen band of the highest-frequency infrared-active STO phonon. The pump electric field was polarized nearly parallel to the [001] crystallographic direction.

**Fig. 2:**
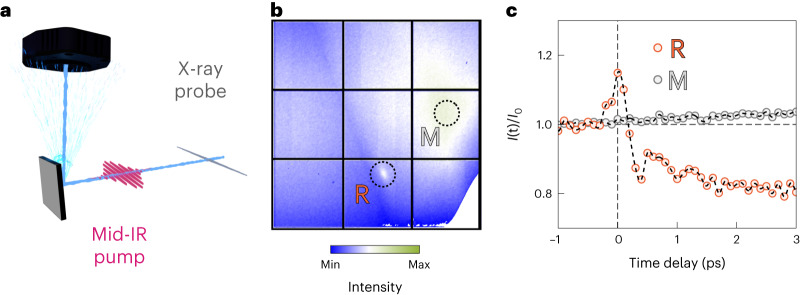
Time-resolved X-ray diffuse scattering. **a**, An intense horizontally polarized mid-IR pulse resonantly excites the highest-frequency STO infrared-active phonon mode along the [001] crystal axis. The vertical diffraction of a time-delayed femtosecond X-ray pulse probes the resulting lattice dynamics in reciprocal space. **b**, Two-dimensional X-ray detector image with selected high-symmetry points R (1/2 1/2 1/2) and M (1/2 1/2 0) at equilibrium. The R point hosts the AFD fluctuations of the cubic-to-tetragonal phase transition. **c**, Measured changes in the X-ray scattering intensity at the R and M points induced by the nonlinear excitation of the crystal lattice. Source data

The X-ray probe pulses of ~50 fs duration were tuned to 9 keV photon energy and spectrally filtered to ~1 eV by a Si(111) monochromator before being focused on the sample. Figure 2b shows a typical image of the diffracted X-rays on a JUNGFRAU pixel-array detector, integrated over 1,000 X-ray pulses. We found localized diffuse scattering at the R point (3.5 2.5 1.5), which was put onto the Ewald sphere as described in Methods, and a broader feature around the neighbouring M point (3.5 2.5 2.0).

Away from Bragg reflections, as is the case here, the diffuse scattering intensity *I*(*q*) at a reduced wavevector *q* = *K* – *G* is proportional to the variance of the atomic displacements 〈*u*_*q*_*u*_–*q*_〉, connected to this wavevector^30^. Here *K* is the total momentum transfer of the scattering process and *G* is the nearest reciprocal lattice vector. At a structural phase transition, the divergence of lattice fluctuations generally results in a critical increase in the diffuse scattering intensity^31^. In the particular case of STO, the cubic-to-tetragonal phase transition at 110 K is driven by the softening and condensation of the AFD phonon mode at the R point in reciprocal space^32^. The increase in lattice fluctuations associated with the onset of this structural transition generates the enhanced diffuse scattering intensity at the well-defined detector region corresponding to the R point^33,34^.

Figure 2c shows the changes in the background-subtracted, integrated scattering intensity around the R point, induced by the resonant excitation of the highest-frequency zone-centre infrared-active phonon at 17 THz transverse-optical-mode frequency. An initial intensity increase is followed by damped oscillations about an overall long-lived reduction in the diffuse scattering signal. Along these dynamics, the coherence length extracted from the width of the scattering profile is enhanced by about 20% during the initial intensity enhancement and reduces by 10% with respect to the equilibrium value at late time delays (Supplementary Section 3). These features were seen to be specific to the R point in reciprocal space, as scattering at Bragg peaks and other points in the Brillouin zone only resulted in small and featureless changes. This is well exemplified by the time-resolved integrated changes measured around the M point (Fig. 2c), where small and slow enhancement was observed.

## Model for nonlinear phonon–phonon coupling

**Fig. 3:**
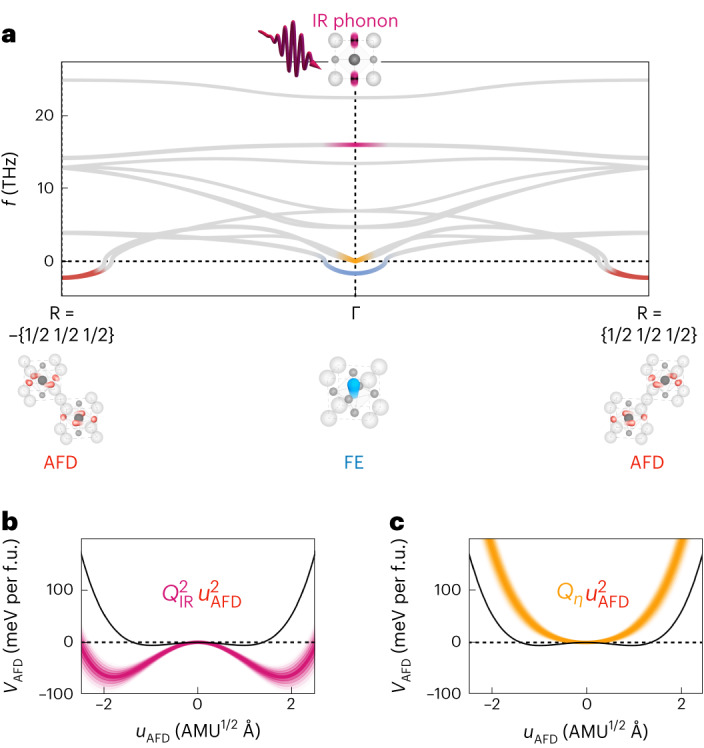
Anharmonic phonon–phonon coupling across the Brillouin zone. **a**, Phonon dispersion of cubic STO (*ϕ* = 0°) along the R−Γ−R direction calculated from an ab initio approach. The coloured lines highlight the transverse-optical phonon branch of the driven infrared-active mode *Q*_IR_ (magenta), strain waves (orange) and the FE soft mode (blue), all at the Brillouin-zone centre. The red-coloured line is the AFD mode at the zone edge. The negative frequencies represent unstable (soft) phonon modes. **b**,**c**, Potential of the R-point AFD distortion as a function of its amplitude *u*_AFD_. The black lines represent the equilibrium potential. The coloured lines show the modifications of this potential due to nonlinear coupling to the zone-centre infrared-active mode *Q*_IR_ (**b**) and to strain *Q*_*η*_ (**c**), as discussed in the main text. Source data

In the following, we discuss a model for these dynamics, which inform further analysis of the data and provide a working hypothesis for light-induced ferroelectricity in STO (ref. ^9^). Figure 3a shows the phonon band structure of the cubic STO phase (*ϕ* = 0°), calculated by an ab initio density functional theory (DFT) approach (Supplementary Section 1). Amongst the other phonon modes, the Brillouin-zone centre hosts the FE soft mode *Q*_FE_ (blue), the acoustic modes related to strain *Q*_*η*_ (orange) and the driven 17 THz infrared-active phonon mode *Q*_IR_ (magenta). The soft phonons connected to the AFD rotation are found at the zone boundary R point*s q* = (±1/2 ±1/2 ±1/2). The unstable symmetry-lowering soft modes manifest themselves as imaginary frequencies in DFT calculations, and are plotted as such. Starting from the resonant excitation of the zone-centre infrared-active mode *Q*_IR_ at its transverse-optical frequency *ω*_IR_, several interaction pathways can be mapped out by utilizing a DFT frozen-phonon approach. Restricting the physics to small AFD rotations (*ϕ* ≈ 0°), reasoned by the experimental setting above the 110 K structural phase transition of STO, three dominant phonon-coupling phenomena are expected in the limit of the third- and fourth-order nonlinearities of the crystal lattice. Bilinear coupling between the phonon eigenmodes is symmetry forbidden, whereas coupling at even higher order becomes relevant only when a larger range of rotation angles *ϕ* towards the tetragonal phase comes into play^35^.

First, the driven *Q*_IR_ mode is expected to nonlinearly couple to a set of acoustic modes to produce strain *Q*_*η*_ (ref. ^9^). The energy of this nonlinear coupling exhibits a square linear dependence on the driven phonon and the strain coordinate, and can be written as ${V}_{{{\rm{IR}}},\eta }={g}_{{{\rm{IR}}},\eta }{Q}_{{{\rm{IR}}}}^{2} {Q}_{\eta }$. Also, because the pulse duration of the mid-IR drive is short compared with the oscillation period of the strain field, which is determined by the ratio of the pump penetration depth and the speed of sound, the strain wave is impulsively launched. For experimentally feasible drive electric fields, we estimate a strain wave with peak values of the order of 0.2% (ref. ^9^).

Second, we expect a coupling of the driven mode *Q*_IR_ to the R-point AFD lattice distortions *u*_AFD_, which—by symmetry—has a biquadratic character ${V}_{{{\rm{IR}}},{{\rm{AFD}}}}={{g}_{{{\rm{IR}}},{{\rm{AFD}}}}Q}_{{{\rm{IR}}}}^{2} {u}_{{{\rm{AFD}}}}^{2}$. This interaction potential implies a parametric excitation of pairs of phonons *u*_AFD_ at opposite wavevectors ±*q* to conserve momentum. This excitation generates lattice fluctuations of the form $\left\langle {u}_{{{\rm{AFD}}}}^{2}\right\rangle$, but no net distortion 〈*u*_AFD_〉 (ref. ^28^). From the ab initio calculations, we find that the coupling matrix element *g*_IR,AFD_ is negative, implying that displacements along the coordinates of *Q*_IR_ soften the AFD mode and hence enhance its fluctuations $\left\langle {u}_{{{\rm{AFD}}}}^{2}\right\rangle$ (Fig. 3b).

Third, we expect that the finite strain *Q*_*η*_ induced at the Brillouin-zone centre via ${V}_{{{\rm{IR}}},\eta }={g}_{{{\rm{IR}}},\eta }{Q}_{{{\rm{IR}}}}^{2} {Q}_{\eta }$ also couples to the R-point AFD distortions *u*_AFD_. This linear square coupling of the form ${V}_{\eta ,{{\rm{AFD}}}}={g}_{\eta ,{{\rm{AFD}}}}\,{Q}_{\eta } {u}_{{{\rm{AFD}}}}^{2}$ has a positive coupling coefficient *g*_*η*,AFD_, and hence, it hardens the AFD mode to reduce its fluctuations $\left\langle {u}_{\rm{AFD}}^{2}\right\rangle$ (Fig. 3c). This coupling counteracts the *V*_IR,AFD_ interaction. Furthermore, the lifetime of strain coupling *V*_*η*,AFD_ to the AFD distortion is determined by the slow relaxation and propagation of the zone-centre acoustic phonons and is far longer lived than the *V*_IR,AFD_ coupling, which is only notable as long as the optical phonon *Q*_IR_ oscillates coherently. The direct coupling of *Q*_IR_ to the FE soft mode *Q*_FE_ is weak, and similar to that shown in another work^9^, we neglect it here (Supplementary Section 1).

To simulate the nonlinear lattice dynamics arising from the physics discussed above, we adopted the approach from elsewhere^36^ and calculated the time-dependent amplitude variance of the AFD distortion $\left\langle {u}_{\rm{AFD}}^{2}\left[t\right]\right\rangle$ induced by the optically driven mode *Q*_IR_[*t*] and strain *Q*_*η*_[*t*]. The coupled system of equations of motion takes the following form.

1 $$\frac{{\partial }^{2}}{\partial {t}^{2}}{Q}_{{\rm{IR}}}[t]+2{\gamma }_{{\rm{IR}}}\frac{\partial }{\partial t}{Q}_{{\rm{IR}}}[t]+{\omega }_{{\rm{IR}}}^{2}{Q}_{{\rm{IR}}}[t]={Z}^{\ast }E[t],$$

2 $$\frac{{\partial }^{2}}{\partial {t}^{2}}{Q}_{\eta }[t]+2{\gamma }_{\eta }\frac{\partial }{\partial t}{Q}_{\eta }[t]+{\omega }_{\eta }^{2}{Q}_{\eta }[t]={g}_{{\rm{IR}},\eta }{Q}_{{\rm{IR}}}^{2},$$

3 $$\begin{array}{c}\frac{{\partial }^{3}}{\partial {t}^{3}}\left\langle {u}_{{\rm{AFD}}}^{2}[t]\right\rangle +2{\gamma }_{{\rm{AFD}}}\frac{{\partial }^{2}}{\partial {t}^{2}}\left\langle {u}_{{\rm{AFD}}}^{2}[t]\right\rangle \\ \,+\,4({\omega }_{{\rm{AFD}}}^{2}+{g}_{{\rm{IR}},{\rm{AFD}}}{Q}_{{\rm{IR}}}^{2}[t]+{g}_{\eta ,{\rm{AFD}}}{Q}_{\eta }[t])\frac{\partial }{\partial t}\left\langle {u}_{{\rm{AFD}}}^{2}[t]\right\rangle \\ \,=-2\left\langle {u}_{{\rm{AFD}}}^{2}[t]\right\rangle \left(2{g}_{{\rm{IR}},{\rm{AFD}}}{Q}_{{\rm{IR}}}[t]\frac{\partial {Q}_{{\rm{IR}}}[t]}{\partial t}+{g}_{\eta ,{\rm{AFD}}}\frac{\partial {Q}_{\eta }[t]}{\partial t}\right).\end{array}$$

The subscripts IR, *η* and AFD denote the frequencies and lifetimes of the *Q*_IR_ mode, strain and AFD distortion, respectively. Also, Z* is the effective charge that couples the infrared-active *Q*_IR_ mode to the external driving field, which we model as a Gaussian pulse centred at the mid-IR pump frequency: $E\left[t\right]={E}_{0}\sin \left({\omega }_{{{\rm{IR}}}}{t}\right){{\rm{e}}}^{-\frac{{t}^{2}}{2{\sigma }^{2}}}$. We determine all the coupling coefficients used in these equations utilizing a first-principles approach on the basis of DFT and adjust a few of them to best match the experimental results (Supplementary Section 2).

**Fig. 4:**
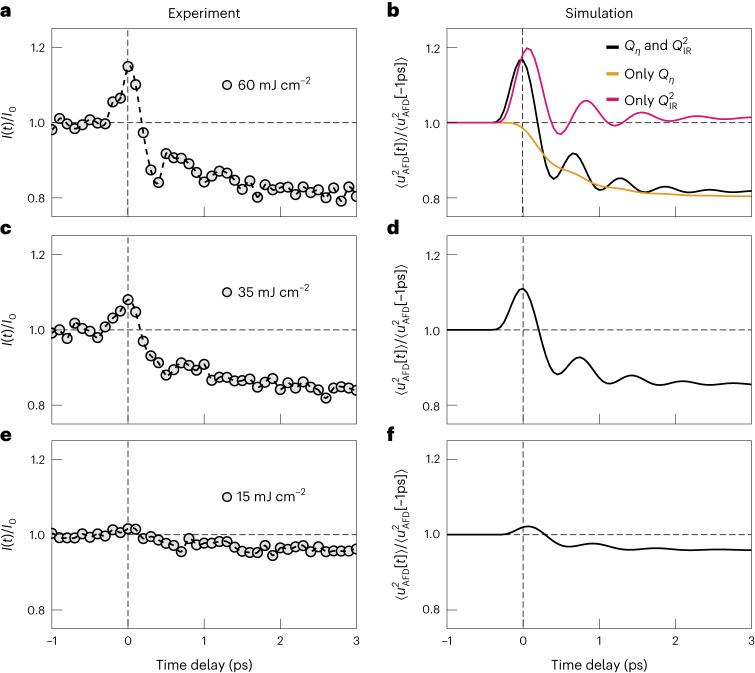
Excitation-fluence-dependent measurements and simulations. **a**, Time-resolved changes in the R-point X-ray diffuse scattering intensity for a mid-IR excitation fluence of 60 mJ cm^−2^. **b**, Simulated normalized changes in the variance in the AFD R-point mode amplitude $\left\langle {u}_{\rm{AFD}}^{2}\left[t\right]\right\rangle$ for the same excitation fluence using the full model presented in equations (1)–(3). The isolated contributions arising from the nonlinear coupling of this mode to the infrared-active phonon *Q*_IR_ (magenta) and strain *Q*_*η*_ (orange) are also shown. **c**, Same measured quantity as in **a** for an excitation fluence of 35 mJ cm^−2^. **d**, Same simulated quantity as in **b** for an excitation fluence of 35 mJ cm^−2^. **e**, Same measured quantity as in **a**,**c** for an excitation fluence of 15 mJ cm^−2^. **f**, Same simulated quantity as in **b**,**d** for an excitation fluence of 15 mJ cm^−2^. Source data

We compare the simulated variance $\left\langle {u}_{\rm{AFD}}^{2}\left[t\right]\right\rangle$ (Fig. 4b,d,f) with the experimentally determined time-dependent R-point scattering intensities (Fig. 4a,c,e), for different excitation fluences. From the simulation, we can isolate the dynamics arising from the different interactions. First, the *V*_IR,AFD_ coupling alone (red curve) results in a picosecond-lived oscillation of $\left\langle {u}_{\rm{AFD}}^{2}\left[t\right]\right\rangle$ at twice the AFD soft-mode frequency as a result of a squeezing of this mode in combination with the short rise time of the *Q*_IR_-mode oscillations. The negative sign of the coefficient *g*_IR,AFD_ leads to a softening of the potential of the AFD distortion, resulting in an initial increase in the variance $\left\langle {u}_{\rm{AFD}}^{2}\left[t\right]\right\rangle$. Next, we simulate the fluctuations of the R-point AFD distortion when coupled only to the optically induced strain via *V*_*η*,AFD_ (Fig. 4b, orange curve). In this case, a slow monotonic decrease in $\left\langle {u}_{\rm{AFD}}^{2}\left[t\right]\right\rangle$ is observed, because the induced strain squeezes the AFD soft mode with a rise time that is too slow to launch oscillations in its amplitude variance. The slow enhancement in the scattering intensity at the M point (Fig. 2c) is of the same origin but with a coupling coefficient of the opposite sign (Supplementary Section 2).

We note that the experimentally observed slow decrease in the R-point diffuse scattering intensity could, in principle, be explained by a transient heating. The increase in temperature would stiffen the (1/2 1/2 1/2) AFD soft mode to reduce the scattering intensity, as observed at equilibrium^34,35^. However, according to temperature-dependent ab initio calculations presented elsewhere^37^, even the M-point AFD mode, which involves octahedral rotations with a (1/2 1/2 0) wavevector, is predicted to stiffen with increasing temperature. Hence, one would expect a similar decrease in the diffuse scattering intensity at the M point in response to a transient heating, which we did not observe in our experiment (Fig. 2c). Hence, the buildup of strain is more probable to induce the long-lived reduction in the scattering intensity.

Taken together, the time-dependent amplitude of the AFD lattice fluctuations $\left\langle {u}_{\rm{AFD}}^{2}\left[t\right]\right\rangle$ is driven by a short-lived phonon–phonon interaction and a longer-lasting strain–phonon interaction. Importantly, the instantaneous shape of the AFD potential also determines the oscillation frequency of the variance $\left\langle {u}_{\rm{AFD}}^{2}\left[t\right]\right\rangle$. Consequently, a larger strain induced by a higher amplitude of the driven *Q*_IR_ phonon results in higher-frequency $\left\langle {u}_{\rm{AFD}}^{2}\left[t\right]\right\rangle$ oscillations. This expectation is experimentally confirmed in the excitation fluence measurements (Fig. 4c,e) and reproduced in the corresponding simulations (Fig. 4d,f).

## Possible implications for light-induced ferroelectricity

**Fig. 5:**
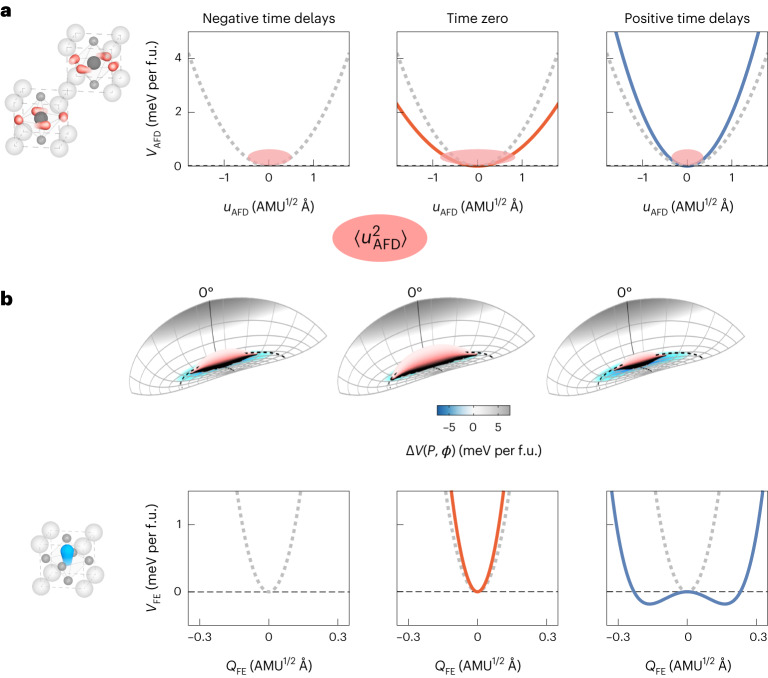
Impact of AFD lattice fluctuations on the potential-energy landscape. **a**, Potential of the AFD R-point mode at equilibrium (grey, negative time delay), with the corresponding variance in its amplitude indicated as the red-shaded area. At zero time delay, the potential softens due to coupling to the resonantly driven infrared-active phonon mode, enhancing the fluctuations $\left\langle {u}_{\rm{AFD}}^{2}\right\rangle$. At positive time delays, the onset of strain hardens the potential, reducing $\left\langle {u}_{\rm{AFD}}^{2}\right\rangle$ fluctuations to below their equilibrium value. **b**, Impact of these dynamics on the potential energy Δ*V*(*P*, *ϕ*), introduced in Fig. 1, in the vicinity of *ϕ* = 0°. At negative time delays, the thermal fluctuations $\left\langle {u}_{\rm{AFD}}^{2}\right\rangle$ cover a region whose averaged potential-energy gain along the FE coordinate is small, thereby prohibiting a condensation of this mode. At zero time delay, the fluctuations $\left\langle {u}_{\rm{AFD}}^{2}\right\rangle$ are enhanced, further reducing the probability to condensate into an FE state. At positive time delays, the $\left\langle {u}_{\rm{AFD}}^{2}\right\rangle$ fluctuations are reduced, favouring condensation of the FE soft mode due to a large averaged potential-energy gain along the FE coordinate. The bottom panel shows the reconstructed potential of the FE soft mode induced by the time-delay-dependent changes in $\left\langle {u}_{\rm{AFD}}^{2}\right\rangle$ and strain. The potential narrows at time zero, but at positive time delays, the combined action of reduced $\left\langle {u}_{\rm{AFD}}^{2}\right\rangle$ and enhanced strain pushes the potential towards the double-well regime. Source data

Having established the AFD dynamics driven in the cubic STO phase, we now discuss their implications on the light-induced ferroelectricity^9^. Our study shows that driving the *Q*_IR_ phonon mode produces short-lived oscillations and initial enhancement in the R-point AFD fluctuations $\left\langle {u}_{\rm{AFD}}^{2}\left[t\right]\right\rangle$, but at longer times, it creates a state in which these fluctuations are suppressed. Figure 5a shows the corresponding modifications in the energy potential of the AFD distortion. The red areas indicate the spread of the fluctuations $\left\langle {u}_{\rm{AFD}}^{2}\left[t\right]\right\rangle$, which dynamically act on the FE distortions in the cubic ground state. This is illustrated in Fig. 5b, which is a zoomed-in view of Fig. 1c at around *ϕ* = 0°. At a negative time delay, fluctuations in the AFD distortions $\left\langle {u}_{\rm{AFD}}^{2}\left[t\right]\right\rangle$ cover a certain width in rotation angle *ϕ*. These fluctuations are expected to suppress the FE state, because AFD rotations away from *ϕ* = 0° reduce the depth of the potential energy along the coordinate of the FE distortion. Then, the excitation of the infrared-active phonon and its biquadratic coupling to the R-point AFD soft mode enhances the fluctuations $\left\langle {u}_{\rm{AFD}}^{2}\left[t\right]\right\rangle$ to suppress the FE state even more. However, the onset of strain distortion at long times after the excitation sizably reduces the AFD fluctuations, such that the phase space—occupied in the FE-energy surface—becomes even smaller than that at equilibrium. In this situation, the condensation of the FE state becomes more probable, and may explain the growth of the FE state. We corroborate this hypothesis in Fig. 5c, where we show the FE soft mode potential in the driven state, calculated from the simulated strain values that match the experimentally induced reduction in $\left\langle {u}_{\rm{AFD}}^{2}\left[t\right]\right\rangle$ at the R point. Indeed, the strain developed at positive time delays induces a double-well potential that is capable of promoting an FE state.

Note that in the experiments reported elsewhere^9^, a light-induced FE phase was observed up to room temperature, that is, both below and above the equilibrium transition into the tetragonal phase. The discussion above only applies to the high-temperature regime (*T* > 110 K), in which paraelectric STO is cubic at equilibrium. For lower temperatures, we expect the same elements discussed above to still be valid, although additional effects could contribute to amplify the photoinduced state.

## Outlook

In summary, we used time-resolved diffuse X-ray scattering and ab initio DFT simulations to clarify the physics of photoinduced ferroelectricity in STO. We show the results that go beyond the measurements of the average position of the atoms in the unit cell captured by Bragg diffraction. We also show how large fourth-order lattice interactions affect the functional response of materials.

Besides possible implications of these findings on the light-induced FE state in STO, we expect consequences for the magnetically ordered fluoride perovskite KMnF_3_. In this compound, which has many common features with the STO, modifications of octahedral rotations at the (1/2 1/2 1/2) R point may substantially affect the exchange interactions between *d* electrons of the Mn^2+^ cations whose spins antiferromagnetically order at the same wavevector^38^. The control of high-order phonon interactions is, in fact, a frontier in the use of nonlinear phononics to manipulate the functional properties of solids.

## Methods

### DFT calculations

We performed first-principles calculations in the framework of DFT to explore the AFD- and FE-energy landscape, phonon excitation spectrum and anharmonic coupling constants of STO within the following technical and numerical settings. In general, we used the Vienna ab initio simulation package (VASP 6.2)^39–41^ to implement DFT and the Phonopy software package for phonon calculations^42,43^. Our computations utilized the pseudopotentials generated within the projector augmented wave method^44^. Specifically, we took into account the configurated default Sr4*s*^2^4*p*^6^5*s*^2^, Ti3*s*^2^3*p*^6^3*d*^2^4*s*^2^ and O2*s*^2^2*p*^4^ potentials and applied the revised Perdew–Burke–Ernzerhof generalized gradient approximation^45^ (PBEsol) for the exchange–correlation potential. As the final numerical setting, after convergence checks, we used a 12 × 12 × 12 Monkhorst-generated^46^ *k*-point-mesh sampling of the Brillouin zone and a plane-wave-energy cutoff of 600 eV. We reiterated the self-consistent calculations until the change in total energy converged up to 10^−8^ eV. All of the phonon mode and anharmonic coupling constant computations were performed with a 4 × 4 × 4 and 2 × 2 × 2 unit cell of cubic STO, respectively. We applied a non-analytical correction to the dynamical matrix to account for the long-range Coulomb interaction^47^.

### Details of time-resolved X-ray diffuse scattering

The X-ray probe beam was sent through a hole bored into an off-axis parabola used to focus the mid-IR beam, enabling collinear excitation and X-ray probing (Fig. 2a). The diffracted X-rays were detected by a JUNGFRAU pixel-array detector, positioned 100 mm from the sample, and normalized to the incident X-ray intensity. The temporal jitter between the pump and probe pulses was monitored using a spectral-encoding technique on a shot-by-shot basis and corrected in post-processing.

The sample was cooled by a cryogenic nitrogen gas jet to approximately 135 K, above the AFD structural transition. To allow for high-excitation fluence at near-normal incidence, we chose a grazing-exit geometry with an exit angle of about 1.5° for R-point scattering. This angle reduces the escape depth of the diffracted X-ray probe to about 1.3 µm, which is shorter than the penetration depth of large parts of the broad mid-IR excitation spectrum. The intensity in the left area of the image (Fig. 2b), separated by the relatively sharp sample horizon, results from air scattering.

**Extended Data Fig. 1:**
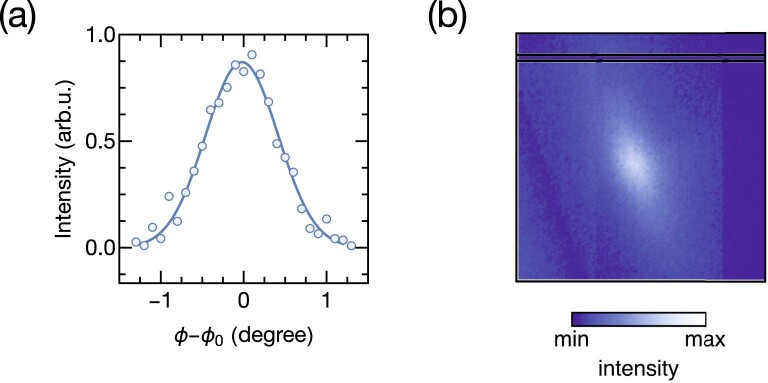
Static X-ray diffuse scattering. Diffuse X-ray scattering from the (3.5 2.5 1.5) R-point with (**a**) showing a rocking curve as a function of sample phi angle and (**b**) a zoom into a background subtracted detector image summed over approximately 200,000 shots of the X-ray free electron laser. Source data

The time-resolved X-ray diffuse scattering experiments were performed with the R point on the Ewald sphere. This was guaranteed by first aligning the sample using a set of Bragg peaks and then optimizing the scattering intensity of the well-defined R-point scattering feature on the detector using a sample *ϕ* scan. Extended Data Fig. 1a shows the result of a rocking curve for the measured (3.5 2.5 1.5) reflection, where we plot the background-subtracted, integrated intensity within the detector region of interest around this point. The width of the intensity profile is about 50 times wider than that of the Bragg peaks in the cubic structure. Extended Data Fig. 1b shows a zoomed-in view into a typical background-subtracted detector image, which was taken in the optimized scattering condition.

Time-resolved changes in the diffuse scattering intensity at the R point (Figs. 2 and 4) were obtained by integrating over the region of interest around the well-defined background-subtracted diffraction spot, and then dividing the intensities of the pumped free-electron laser shots by the intensities of unpumped free-electron laser shots. This analysis of the integrated intensity allowed us to trace the amplitude of the $\left\langle {u}_{\rm{AFD}}^{2}\left[t\right]\right\rangle$ lattice fluctuations as a function of time delay after mid-IR excitation.

## Online content

Any methods, additional references, Nature Portfolio reporting summaries, source data, extended data, supplementary information, acknowledgements, peer review information; details of author contributions and competing interests; and statements of data and code availability are available at 10.1038/s41563-023-01791-y.

### Supplementary information

Supplementary Information Supplementary Sections 1–3, Figs. 1–7, and Tables 1 and 2.

Supplementary Data Structure of the cubic STO unit cell utilized in this study.

### Source data

Source Data Fig. 1 Potential-energy data plotted in Fig. 1c,d.

Source Data Fig. 2 X-ray diffuse scattering data plotted in Fig. 2b,c.

Source Data Fig. 3 Phonon dispersion data plotted in Fig. 3a, and potential-energy data plotted in Fig. 3b,c.

Source Data Fig. 4 R-point diffuse scattering intensity data plotted in Fig. 4a,c,e, and the simulation data on variance in R-point amplitude plotted in Fig. 4b,d,f.

Source Data Fig. 5 Potential-energy data plotted in Fig. 5.

Source Data Extended Data Fig./Table 1 Diffuse scattering X-ray data plotted in Extended Data Fig. 1.

## Data Availability

Source data are provided with this paper. Further datasets collected for this study are available from the corresponding author on request.
