# Supplementary material for: Quenched lattice fluctuations in optically driven SrTiO3
Source: Nat Mater. 2024 Feb 1;23(3):363–8. doi: 10.1038/s41563-023-01791-y (PMC10917662; doi:10.1038/s41563-023-01791-y)
Supplement: Supplementary file 1 — Supplementary Sections 1–3, Figs. 1–7, and Tables 1 and 2. [file 41563_2023_1791_MOESM1_ESM.pdf]

nature materials

Article https://doi.org/10.1038/s41563-023-01791-y

Quenched lattice fluctuations in optically driven $SrTiO_3$

In the format provided by the authors and unedited

Table of Contents

S1) Density Functional Theory Calculations 2

S2) Calculations of the light-induced lattice fluctuations 4

S3) Time-dependent changes of the coherence length of the AFD lattice fluctuations......... 7

References Supplementary Information 8

S1) Density Functional Theory Calculations

The starting point of our first-principle investigation is the cubic unit cell of $SrTiO_3$. First, we determined the DFT equilibrium volume for this cell, which is $V_{cell} = 59.2\ \text{Å}^3\ (a = 3.898\ \text{Å})$. We next calculated the phonon band structure, shown in Fig. S1, for selected points and their connecting lines in reciprocal space. Here, we plot unstable modes, which have an imaginary frequency, with negative frequencies. We found the ferro-electric instability at the zone center Γ point and the antiferrodistortive instability at the zone edge R-point. We found a second antiferrodistortive mode at the M-point, which in contrast to the R-point soft mode involves *in-phase* rotations of all the octahedra and is stable. While our calculations were performed at T = 0 K, their temperature dependence was recently computed in Ref. 1.

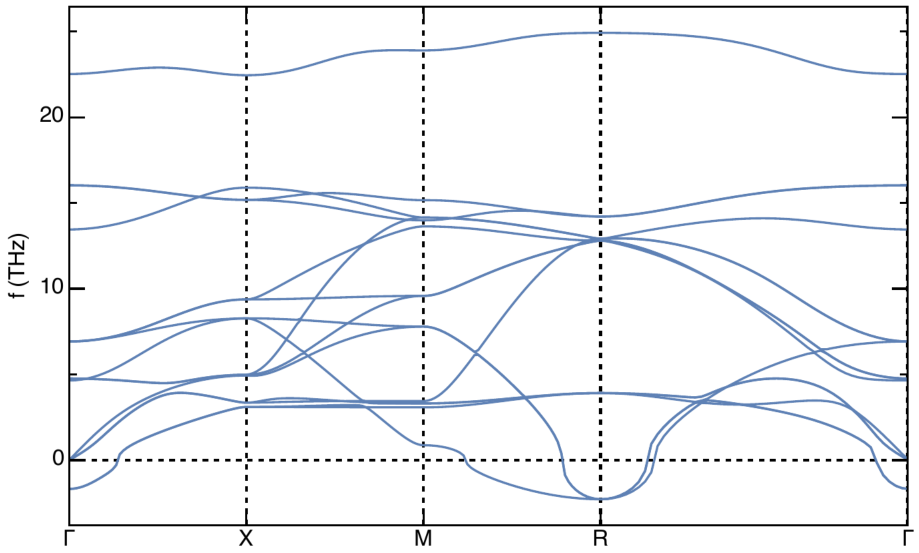

Figure S1. | The phonon band structure of $SrTiO_3$ at T = 0 K, obtained from first-principal calculations described in the text.

Next, we determined the strengths of the anharmonic couplings using a frozen-phonon approach. We first modulated a 2x2x2 unit cell of $SrTiO_3$ with a superposition of two eigenmodes and then fitted the resulting energy landscape by an appropriate polynomial form as given in the main text. We are specifically interested in nonlinear phonon couplings that involve combinations of the driven zone-center Q_{IR} mode and the modes at the R- and M-points and additionally considered the coupling of

tensile stress at the Γ-point to both these zone boundary modes. Figure S2 shows the result of the computations as a function of the amplitudes of the Q_{IR} mode and tensile strain Q_η.

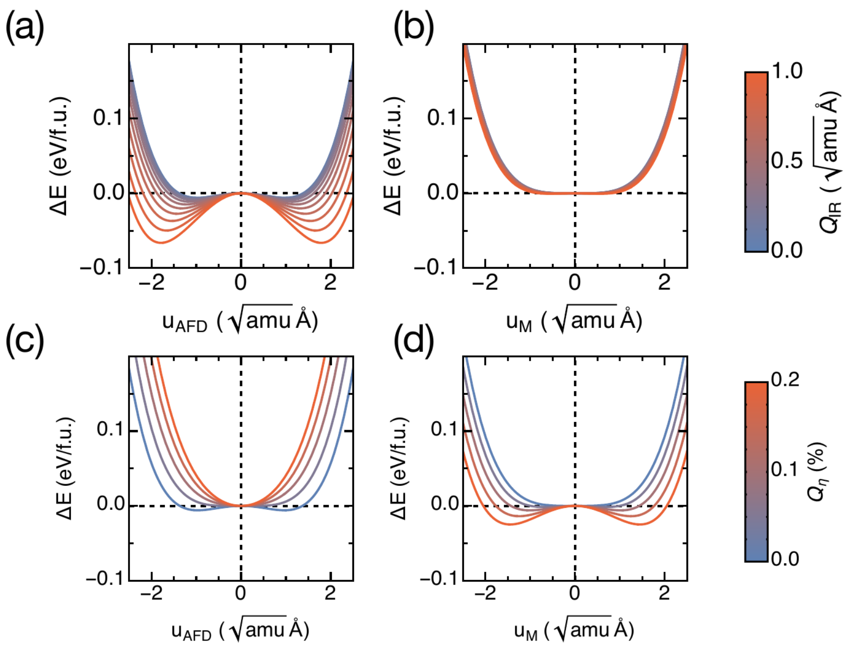

Figure S2. | The anharmonic potentials of the antiferrodistortive mode u_{AFD} at the R-point and of the in-phase rotational mode u_M at the M-point, both as a function of the driven zone-center Q_{IR} mode (a,b) and strain Q_η (c,d).

Table S1 reports the nonlinear coupling coefficients fitted to these calculations. The coupling coefficients of the Q_{IR} mode to the Brillouin zone center ferroelectric soft mode and to tensile strain, represented by an acoustic mode Q_η, obtained by the same procedure, were previously published in Ref. 2 and are listed here for completeness.

i	u_i^2 [meV/(amu Å)]	u_i^4 [meV/(amu Å)^2]	$u_i^2 Q_{IR}^2$ [meV/(amu Å)^2]	$u_i^2 Q_\eta$ [meV/(amu$^{1/2}$Å %)]
AFD	−12.36	6.49	−29.10	2.32
M	0.51	5.36	−4.02	-1.21
FE	-11.82	76.35	1.55	-3.38

Table S1 | Fitted harmonic/anharmonic constants and coupling coefficients for the modes at the R-, M- and Γ-point in reciprocal space. We abbreviate the atomic mass unit with amu.

Lastly, we performed total energy calculations to fully map the energy lowering of the ferroelectric state as a function of the R-point antiferrodistortive rotation (see for example Ref. 3). In Figure S3, the

dashed line walks along the minimum in ferroelectric energy, and the corresponding values in the ferroelectric polarization correspond to the $P_0(\phi)$ polar plot in Fig. 1 of the main text.

Our experiment was performed in the cubic $SrTiO_3$ state ($\phi = 0°$). Light-induced nonlinear changes in lattice fluctuations then take place in a very small regime around this angle where fourth-order mode coupling terms are sufficient to describe the dynamics. The behavior for larger angles is the result of higher-order coupling terms, as shown for similar compounds in Ref. 4.

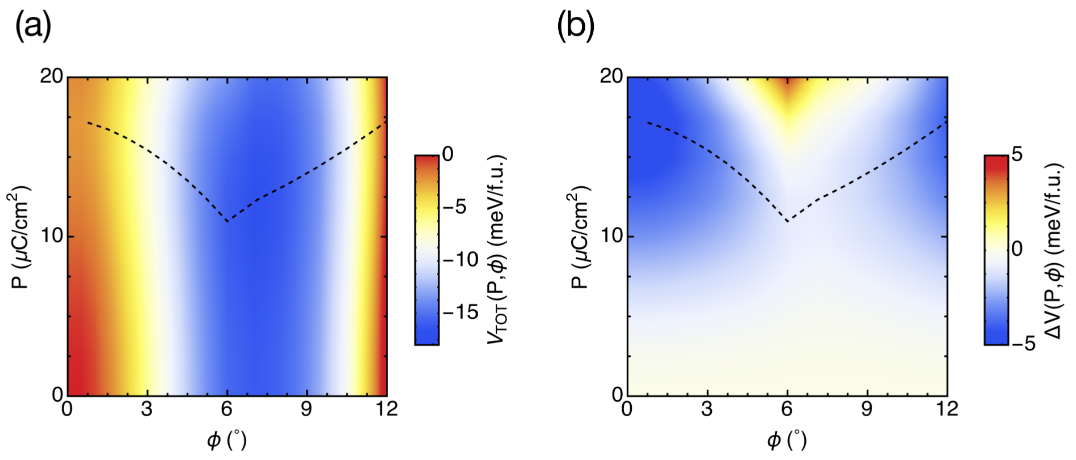

Fig S3 | Ferroelectric energy landscape of $SrTiO_3$ computed from first-principles calculations. (a) DFT total energy $V_{TOT}(P,\phi)$. (b) Potential energy landscape $\Delta V(P,\phi) = V_{TOT}(P,\phi) - V_{TOT}(0,\phi)$. Dashed lines indicate the polarization $P_0(\phi)$, which is also shown in the polar plot in Fig. 1 of the main text.

S2) Calculations of the light-induced lattice fluctuations

We used Equations (1)-(3) of the main text to calculate the dynamics of the lattice fluctuations upon driving the Q_{IR} mode at different excitation field strengths. The starting point for all the phonon parameters of this procedure were the first-principles calculations described in Section 1. Eigenfrequencies of the phonon modes were taken from experimental data presented in Ref. 5, as DFT only provide values for the unstable system at zero Kelvin. Moreover, the starting value for the frequency of the strain field Q_η was taken from the ratio of the penetration depth of the mid-infrared excitation pulses and the sound velocity[6], giving a frequency of in the GHz range. In this sense, Q_η

represents a collection of acoustic modes and consequently the interpretation of its eigenfrequency and lifetime is not to interpreted as that of a single vibrational mode.

A few of the calculated phonon parameters, including their damping coefficients, were then adjusted within certain physically reasonable boundaries to best match the solutions of these equations to the measured changes in diffuse x-ray scattering. Specifically, we constrained the signs of the coupling coefficients to the DFT values, restricted the frequency of the strain to the GHz range and confined the maximum allowed strain to a value below 0.3 %. Lastly, we also allow a variation of the eigenfrequency of the R-mode within the +/- % Kelvin tolerance of the experimentally determined temperature. Using these conditions, we concluded with the calculated lattice fluctuations $\langle u_{AFD}^2[t]\rangle$ shown in Figure 3 of the main text. The corresponding phonon coefficients are reported in Table S2.

$\boldsymbol{f_{IR}}$<br>16 THz	$\boldsymbol{\gamma_{IR}}$<br>1.4 THz	$\boldsymbol{g_{IR,\eta}}$<br>$-0.05\ \mathrm{THz}^2/(\sqrt{\mathrm{amu}}\text{Å})$
$\boldsymbol{f_{\eta}}$<br>0.5 GHz	$\boldsymbol{\gamma_{\eta}}$<br>1.0 THz	$\boldsymbol{g_{IR,AFD}}$<br>$-23.31\ THz^2/(\mathrm{amu}\ \text{Å}^2)$
$\boldsymbol{f_{R}}$<br>$0.6-0.7$ THz	$\boldsymbol{\gamma_{R}}$<br>1.4 THz	$\boldsymbol{g_{\eta,AFD}}$<br>$4487.77\ \mathrm{THz}^2/(\sqrt{\mathrm{amu}}\text{Å})$

Table S2 | Parameters obtained from the best match of the solutions of equations (1)-(3) to the experimental data.

To confirm the calculated negative sign of the coupling coefficient $g_{IR,AFD}$, Figures S4 a,b show the comparison of the calculated lattice fluctuations $\langle u_{AFD}^2[t]\rangle$ for a positive and negative sign of the coupling coefficient. The initial rise of the lattice fluctuations oscillations at zero time delay is obtained only for a negative coefficient. A similar case can be made for the sign of the strain-AFD coupling coefficient $g_{\eta,AFD}$.

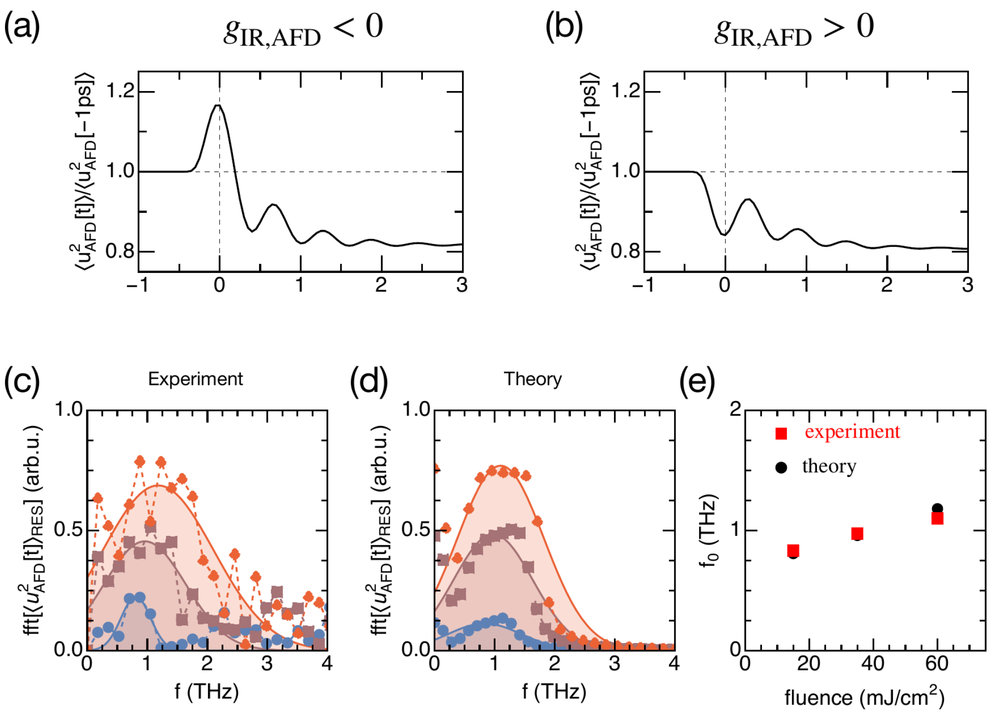

Figure S4. | Calculated fluctuations $\langle u^2_{AFD}[t]\rangle$ of the R-point antiferrodistortive lattice distortions for positive (a) and negative (b) coupling coefficients of $g_{IR,AFD}$. Panels c,d show the Fourier transforms of the oscillatory contributions to the changes in R-point diffuse scattering intensity and to the calculations shown in Figure 3 of the main text, both for excitation fluences of 15, 35 and 60 mJ/cm². Solid lines are Gaussian fits to these data. (e) Comparison of the excitation fluence dependent peak frequencies obtained from Fourier transforms plotted in c,d.

Furthermore, we compared the frequency components of the calculated and measured R-point lattice fluctuations $\langle u^2_{AFD}[t]\rangle$. To this end, we performed Fourier transformations of the oscillatory components to experimental and theoretical curves, achieved by subtracting the slowly varying contributions of the strain induced change to the lattice fluctuations. The resulting excitation strength dependent Fourier spectra are plotted in Figure S4 c,d together with Gaussian fits to these data. We found an increase in the frequency of the diffuse scattering intensity oscillations and of the calculated $\langle u^2_{AFD}[t]\rangle$ oscillations as a function of fluence, confirming that higher excitation fluences result in enhanced strain that modulates the potential energy of the AFD distortion.

Lastly, using the same approach, we performed simulations of the time dependent lattice fluctuations $\langle u^2_{M}[t]\rangle$ at the M-point. Since the escape depth of the x-rays scattered with a wave vector connected to the M-point is a few times the penetration depth of the mid-IR excitation pulses, we only provide a qualitative comparison, shown in Fig. S5. Since the signs of the M-point coupling coefficients $g_{IR,M}$ and

$g_{\eta,M}$ exhibit opposite signs than the R-point coupling coefficients $g_{IR,AFD}$ and $g_{\eta,AFD}$, we observe a sign change in the dynamics of the lattice fluctuations. This is confirmed in the experimental data shown in Figure 4c of the main text.

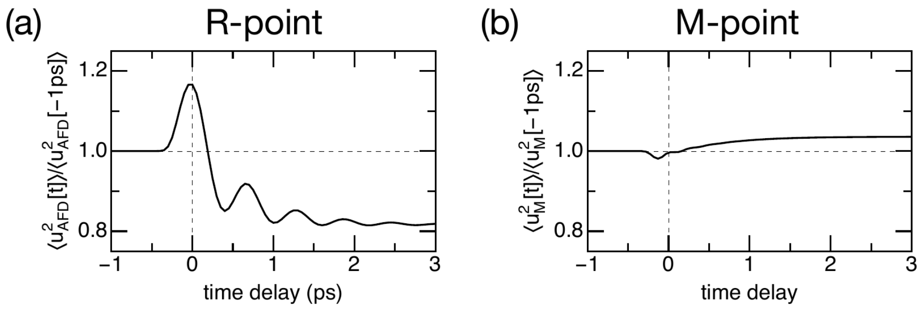

Figure S5. | Calculated fluctuations $\langle u_{AFD}^2[t] \rangle$ of the R-point antiferrodistortive lattice distortions and $\langle u_M^2[t] \rangle$ of the M-point lattice rotations, both for the highest excitation fluence of 60 mJ/cm².

S3) Time-dependent changes of the coherence length of the AFD lattice fluctuations

We analyzed transient changes in the correlation length of the AFD lattice fluctuation by inspecting the shape of the time dependent scattering intensity profiles. Figure S6a shows the scattering intensity profile along the diffraction spot's long axis at negative time delay (blue), close to time zero where the diffuse scattering intensity is maximum (red), and at a long positive time delay (green). This axis lies parallel to the sample horizon so that the x-ray photon escape depth is constant along this cut to avoid mixing any penetration depth effects into the analysis. The time-dependent scattering amplitude extracted from the Lorentzian fits to the data (solid lines), shown in Fig. S6b, reproduces well our analysis obtained by integrating the detector images over the region of interest (see Figure 2c of the manuscript), here with a lower time resolution as we binned the data over 0.3 ps to reduce the noise. Beyond, the width of the scattering intensity profile, shown in Fig. S6c, narrows by about 20% around time zero before it broadens by about 10% at long delays, both relative to the equilibrium. While these changes are relatively small, they show that at early times not only the amplitude of the R-point lattice fluctuations, but also their coherence length, increases – implying that the $Q_{IR}^2 u_{AFD}^2$ coupling

instantaneously stimulates the transition to the tetragonal phase. The slower $Q_{\eta}u_{AFD}^{2}$ strain driven dynamics reverse this effect by not only reducing the amplitude of the lattice fluctuations, but also the characteristic lengths over which they occur.

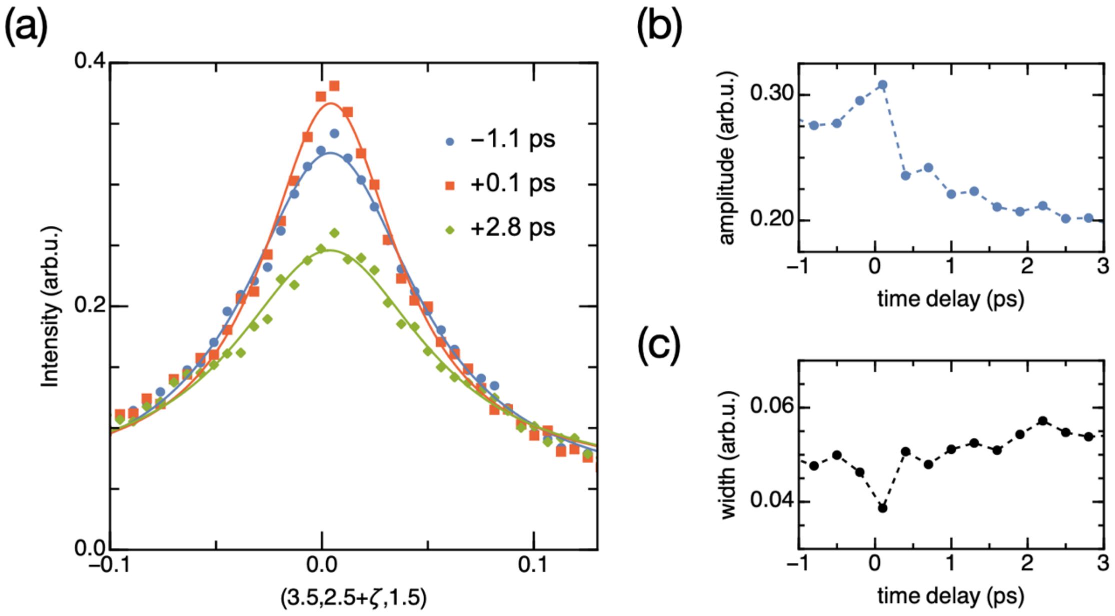

Figure S6. | Time dependence of the scattering intensity profile at the (3.5 2.5 1.5) R-point. (a) Scattering intensity profile along the diffraction spot's long axis at three different pump-probe time delays (data points), together with fitted Lorentzian functions (solid lines). (b) Extracted amplitude. (c) Extracted width.

<u>**References Supplementary Information**</u>

1. Verdi, C., Ranalli, L., Franchini, C. & Kresse, G. Quantum paraelectricity and structural phase transitions in strontium titanate beyond density functional theory. *Phys Rev Mater* **7**, L030801 (2023).
2. Nova, T. F., Disa, A. S., Fechner, M. & Cavalleri, A. Metastable ferroelectricity in optically strained SrTiO3. *Science* **364**, 1075–1079 (2019).
3. Aschauer, U. & Spaldin, N. A. Competition and cooperation between antiferrodistortive and ferroelectric instabilities in the model perovskite SrTiO3. *Journal of Physics: Condensed Matter* **26**, 122203 (2014).
4. Gu, T. *et al.* Cooperative Couplings between Octahedral Rotations and Ferroelectricity in Perovskites and Related Materials. *Phys Rev Lett* **120**, 197602 (2018).
5. Shirane, G. & Yamada, Y. Lattice-Dynamical Study of the 110°K Phase Transition in SrTiO3. *Physical Review* **177**, 858–863 (1969).
6. Bell, R. O. & Rupprecht, G. Elastic Constants of Strontium Titanate. *Physical Review* **129**, 90–94 (1963).
